# Urban Networks, Micro-agriculture, and Community Food Security

**DOI:** 10.1007/s43615-021-00117-x

**Published:** 2021-11-18

**Authors:** Sarah N. Gatson, Marissa Cisneros, Robert Brown, Jacqueline A. Aitkenhead-Peterson, Yu Yvette Zhang

**Affiliations:** 1 Department of Sociology, TAMU 4351, Texas A&M University, College Station, TX 77843-4351 USA; 2 Veterinary Physiology & Pharmacology, Texas A&M University, College Station, TX USA; 3 Landscape Architecture & Urban Planning, Texas A&M University, College Station, TX USA; 4 Soil & Crop Science, Texas A&M University, College Station, TX USA; 5 Agricultural Economics; Energy Institute, Texas A&M University, College Station, TX USA

**Keywords:** Community food security, Food sovereignty, Urban agriculture, Regenerative systems

## Abstract

The white paper first outlines the state of inequity in food security/sovereignty in our area of focus, taking into account historical context as well as emerging and ongoing effects of the COVID-19 pandemic and community and policy responses to it. We then discuss a food acquisition intervention, structured as a longitudinal, collaborative research, and service-learning effort known as Everybody Eats. The white paper provides detailed discussion of competing understandings of agriculture, horticulture, and the social problem of food insecurity; the preliminary data that has led to a current collaborative effort to enhance the skillset of people previously not understood as food producers and provisioners, but only as end-user consumers; and the new iteration of the project wherein specific sets of expertise from diverse disciplines are deployed both to offer a more robust intervention, and bring new methodologies to bear in assessing the ecology of a local foodshed. We propose mobilizing existing resources and expertise of the Land Grant/Cooperative Extension system to act as a regional hub for facilitating full community food security (caloric and nutritional adequacy) and food sovereignty (participatory decision-making regarding living spaces and culturally appropriate foodways). Finally, we illustrate how a nexus of faculty, working from a service-learning advocacy perspective and embedded in a participatory action framework, provides a mechanism for bringing together and sustaining a community of intellectually diverse researchers and stakeholders.

## Introduction

Community food security has been severely impacted during COVID-19 as various networks of food resources were disrupted by business modifications, school shut-downs, shifts in locations of eating, etc. As well as affecting everyday business, these shifts negatively affected existing policy aimed at mitigating community food insecurity specifically. The policies that assist communities with low food security consist of a patchwork of aid. The Supplemental Nutrition Assistance Program (SNAP) assists families with purchases of food. The same eligibility standards govern public school meals, and the Women, Infants, and Children (WIC) nutrition program. Other public-private partnerships govern community aid resources such as food banks and pantries. However, with the COVID-19 outbreak, the accessibility of each of these resources has seen further restrictions. While prevalence of US food insecurity in 2019 fell below the 2007 pre-Great Recession level for the first time [1], Ziliak [2] found that food insufficiency during the COVID-19 pandemic increased threefold compared to 2019.

The intersecting economic and policy landscape reflects a glaring gap in food security, as community members with access to private bank accounts and credit cards have transitioned to online ordering and pick-up and delivery services far more easily than those that must rely on cash, in-kind donations, and SNAP or WIC funds which are distributed electronically. Although some states now allow application of SNAP benefits to online grocery sites, these preliminary steps have only been implemented by a few businesses. Even if these experiments were expanded, extra charges such as delivery and service fees are an additional cost that further taxes overstretched budgets. However, local collective and flexible responses to community food aid have showed positive outcomes. For instance, schools and community organizations extended provision of free meals to families in need. Addressing equity, some districts offered food delivery in addition to pickups. Some communities initiated their own food networks: modifications of networks such as meals-on-wheels, pop-up pantries, and direct-to-consumer coalitions, as well as garden food shares.^1^

## Pressing Societal Need

Resolving community food insecurity requires longitudinal data-gathering at the micro- (individual, household, and neighborhood), meso- (organizational and institutional), and macro- (policy, law, and political economic) levels, with particular attention paid to the holistic and historical ties and ecological contexts where humans reside [4]. That there is enough food, fertile soil, and potable water but that there is not enough equitable access to these basic human needs is arguably the fundamental pressing societal need of the current historical moment.

## Potential of Transformative Convergence Research

The holistic and multilevel analyses required to address the societal need of our focus requires an interdisciplinary lens and a collaborative team. Expertise across a range of methodologies, methodological tools, applied models and interventions, and explanatory/theoretical frameworks is needed. As well, expertise in the experiences and behaviors of human and non-human actors is also required. Since we operate from an ecological framework that understands humans as embedded in an ecological system, focus on the soil microbiome, plant and animal life cycles, food and supply chains, and meaningful human community and participatory citizenship is what will lead to an empowered resolution to fulfilling community food security needs.

## State of the Science — Key Challenges

The global question we focus on is how boundaries and power impede the meeting of humanity’s most basic needs. While aspects of the larger project examine the microlevel acts involved in food preparation and consumption, herein, we zero in on the arenas of provisioning, and the ways in which access to time and space facilitate provisioning. Thus, we begin from assessing how these needs are/are not met from the microlevel outwards. We take our cues from Marjorie DeVault’s classic *Feeding the Family: The Social Organization of Caring as Gendered Work* [5]. Therein, DeVault assesses everyday household foodways, and breaks down the daily reproductive work of feeding household members into task areas: enacting family meals, provisioning, and the cultural ordering of time and space.

Published in the early 1990s US context, DeVault engages with research subjects in their homes regarding food-focused household labor in urban areas, and thus conceptualizes provisioning largely as the processes involved in acquiring food from conventional grocery stores. DeVault emphasizes that she calls this “provisioning rather than shopping…to indicate that there is more to it than we can see inside a store, and to emphasize its embeddedness in a socially organized household practice” [5].^2^

In the USA, food access and provisioning in particular has been cemented — both conceptually and structurally — around the supply chain that begins in large-scale agriculture and ends in the conventional supermarket [7–9]. Prior to this large-scale, industrial, hegemonic provision supply chain, agriculture in the USA was deeply predicated on two simultaneous strands of dispossession and barring particular communities from land and other ecological resources. On the one hand, non-sustainable political-economic and agricultural practices and products were violently enforced onto indigenous communities [10]. On the other, enslaved Africans and their descendants were the uncompensated and uncredited backbone of both small-scale and large-scale, plantation agriculture, the foundational economic engine of American settler colonialism [11–15].

The dispossession of, and lack of access to, the sort of arable land that could produce an abundant food security, indeed food sovereignty, is not merely a product of a contested colonial past, rather it is ongoing [8, 16]. Food sovereignty — in contrast to/alongside of food security — aims to alleviate not only the inequitable distribution of food, but the deliberately unheard voices of food producers and food consumers in food policy specifically pertaining to food production [17, 18]. It is an aim to democratize the food system [17–20]. Such a paradigm considers the drastic break between the vast majority of people, and a direct experience of being in relation to land. This break results in the majority of people not being cognizant of land as an ecological whole: soil, water, and air. As a result, we see a disconnect of understanding land as being the constitutive foundation of human existence. This disconnect simultaneously creates a space of food alienation, which interacts with both dispossession and food insecurity. Here, lies the opposite of food sovereignty, which considers the connection to, and access of, land as well as community interrelations and production of food. Food alienation, then, is the disconnect from community, land, and the realities of interdependence of food creation both at the individual, community, and policy level. This alienation creates a space wherein each household views food as an individual-level problem of procurement, largely dependent upon policies and a food environment wherein household members have a near-complete disconnect and where their experiences of provisioning are not centered in the policy-making arena. However, also ever-present historically and today are resilient pockets of resistance and purposeful practitioners of non-dominant cultures of agronomy and ecological epistemologies [6, 14, 18–26].

In discussing where we should look to understand realities of food insecurity and food alienation, Pionetti argues, “Poverty is, indeed, a matter of ‘capacity deprivation’, a notion which refers to the lack of real opportunities in choosing a particular livelihood or type of living” [24]. Putting power and equity at the center of our framework, we assert the need to focus on, “Food, its scarcity, the desire and opportunity to grow it, and the need to do it in ways that are appropriate to place and circumstance” [27]. In doing so, we view agriculture as existing in a legal and political nexus wherein which concepts of property, residence, and consumption converge in contrasting and contested visions of what counts as ‘the good life’ for a citizen of the USA. These lead to the questions: Who has access to the land, and the livelihoods that could be produced from it [18, 23, 28]? How is that access granted/taken up at the microlevel as households enact their livelihoods in the context of macrolevel policies that are implemented by meso-level organizations [29]?

To begin answering these questions, we must consider the dueling policies and approaches to farm and garden. While these two concepts have a long history in the production of food in the USA, the governmental approaches, and meso-level interactions, of these two structures are at odds.

### Where the Farms Are?: the Colonial Enterprise of the Land Grant

From its beginnings, Cooperative Extension has conceptually separated agriculture into farming and gardening and separated agriculture from home economics (relabeled family and consumer sciences in 1999). This separation has largely remained despite the fact that the World War II gardening programs, whose oversight was relegated to women — home economics agents and 4-H leaders within Cooperative Extension, provided “more than forty percent of the vegetables grown for fresh consumption” nationally in 1943 [9]. In examining this separation, we see that alongside the near monopoly of large-scale agriculture exist the programs of Cooperative Extension (which fall under the partial purview of the United States Department of Agriculture [USDA]). These programs, which focus on gardens and small farms that lead to community food security, have ebbed and flowed since their inception in 1915. Further illustrating the separation between farm and garden are standardized conceptions of farm dimensions. Farm “size” is defined in terms of gross sales or income, not acreage, and has changed over time. In the late 1980s, a farm with an income of $40,000 or less was defined as “non-commercial” by the USDA [9]. Currently, the USDA threshold for “non-commercial status is less than $10,000 in gross sales [30]. To be classified as a farm at all, one’s agricultural activities must involve “enough land or livestock to generate $1000, whether or not actual sales reach that level.” [Ibid] The USDA goes further, zeroing in on engagement with the market rather than on the variety of productive tasks engaged in, “Most of these operations are better described as rural residences; the households on these farms — and on many other small farms — rely heavily on off-farm income.” [Ibid]

Putting an exclusively (and nearly exclusively large) for-profit market definition onto the word “farm” makes nearly inevitable that a gender dichotomy will be overlain onto the tasks and activities that are involved in bringing food from the soil to the table [31]. This is the case even though everyone on a subsistence farm is a “provider”, and the bodies that engage in the array of tasks needed overlaps gendered notions of appropriate work. Further, if we put those tasks first into the categories of what is actually done (i.e., raising animals for meat; raising animals for byproducts), rather than by their already implicitly or explicitly gendered nomenclature (i.e., “keeping chickens”; “being a milkmaid”), the cultural overlay becomes explicit.

Although both are verbs, to produce and to consume take on connotations of gender that render their meanings into a dichotomous, mutually exclusive pair of masculine and feminine characteristics: men produce/provide and are active political economic agents; women consume and are passive, lower-level economic managers at most, and dependent domestic engineers at least. Of course, this is fundamentally a false dichotomy [31]. In the separated programming of Cooperative Extension historically, we had actual producers (usually seen as skilled at turning raw materials/ingredients into value added products, not as producers of the raw materials themselves) cast as dependent consumers if they were women doing it inside a household/for a family — even if their products were later sold to support the household at a local market. Money could thus be understood as provision (in the purview of men) versus money as budget (in the purview of women). Boys were in corn clubs, classified as learning to farm for markets; girls were in tomato canning clubs — even though they grew the tomatoes they canned [9]; this was not understood as a “true” economic activity, but rather home (private) economics. The same agriculturally productive, and household reproductive, activities are thus neatly conceptually segregated and taken-for-granted going forward as proper social roles,

The better farm management programs took account of the farm women and indeed of the farm family in determining what could and should be done on a particular farm. Farm women traditionally maintained the household and cared for the children, but a survey made in 1920 by the Office for Extension Work in the North and West showed that farm women also made contributions to the economic well-being of the farm. Some 85% cared for chickens, 25% for livestock, and 56% for gardens; 36% milked cows; 33% made butter to sell; and 24% engaged in fieldwork for an average period of seven weeks. About 1/3 of the women kept farm accounts. About 79% of the women used kerosene lamps, 61% carried water from an outside well, and most cooked on kitchen ranges and heated the house with stoves burning wood or coal [9].

The above passage contains one of the best examples of the seamless acceptance of the everyday operation of gendered labor — the term “farm women,” not farmers. We see the same sort of separation of actual labor (intellectual and manual) from socioeconomic status in the use of “slave” and “migrant laborer” when discussing people engaged in agricultural work. To be a farmer is, first and foremost in the USA’s actual organizational and structural practice, to be counted as the owner of land.

In approaching our research, we consider these above framings to be centered on the valuation and devaluation of bodies in relation to food production. We must question who we as a society understand to be a food producer, and who a food consumer, and the dichotomy between the two must be made explicit. Further, at every level of policy implementation, we must also interrogate the historicized legal policies at play in the maintenance of these conceptions, and how they affect the lives of individuals in micro and meso-level interactions [4]. With such considerations in mind, we must act to produce greater food sovereignty and reduce food alienation.

## Data and Knowledge Gaps

We live where we work, and we must confront our relative positions of power and agency and strive towards transparency. Given our field in academia, our research objectives, and the relative situation of both the meso- and macro-level power structures, we must be reflective of how our involvement with the community as researchers could aid as well as harm [20, 32]. Moreover, our involvement is not truly with the community, but with *our* community. Similar to the above dichotomy of farmer and gardener, so too is the dichotomy of researcher and participant. However, in studying community food security, are we the researchers not also consumers of food? In the procurement of food, do we not interact with the same community we study?

To study a community, we must understand the intricacies of power that tacitly lay beneath the surface as they touch not just the policy within that community, but also everyday interactions (including rhetoric and discourse), placement of groups of people, access, and trust. Furthermore, when studying our community, we must be aware of where we stand within said community, and how that further impacts comfort and trust. We thus have to consider the impact and place of engaging with issues of community food security in the midst of climate change whilst living in the same ecological foodscape as our research participants. To take all these relations into consideration, we first consider the system in which we reside and work from.

The Land Grant system and its interface with the USDA and Cooperative Extension form the structural apparatus within which the ecological community that is our focus — the Brazos Valley of Texas — exists. The particular landscape that is Texas A&M University and its surrounding community, and its interface with agriculture, horticulture, and various sectors of the food movement is where our data will be co-produced with members of that community. Our methodological process will be an attempt to expose the ecological, economic, design, and sociolegal terrains that make participation in direct food production feasible, and examine the questions of what makes a farm, a farmer, and food security/insecurity and food sovereignty/alienation.

We understand community food security and community food provision networks (including conventionally understood market supply chains) as complex, nested relationships, and the core service our collaborative team is working to facilitate is the growth and production of vegetables and fruits where people reside — the direct participation of anyone in food agriculture. This has included portable herb gardens, container gardens, and a variety of raised bed configurations. The team includes novice and experienced undergraduate students, graduate students, and collaborating faculty members (including the authors herein) that work as small teams at a variety of community sites and residential locations. They act to facilitate holistic community food security through the reinvigoration of distribution networks of past Cooperative Extension programs.

We use the methods of participatory action ethnographic research (participant observation, interviews, surveys, historical and archival analyses, and quantitative analyses using large datasets). In line with Matarrita-Cascante, Sene-Harper, and Ruyle’s community-driven program framework [33], we approach our work primarily as research of our community, meaning that the resources are primarily owned by participants. However, understanding the power relations that are involved with our community, specifically the university/non-university divide, we have created various community ties in order to include many non-professional/non-academic voices in our project development and implementation. We aim to create increased community food security through analysis of access to food and food production itself, as well as a myriad of mechanisms that make such access possible: social capital, trust, capacity, support, and empowerment [33]. Overall, this framework considers the community as an invested partner in research that aims to produces food equity, as well as conserve the land wherein we produce said food.

Our purpose in our collaboration is to introduce and facilitate sustainable and regenerative residentially based food production techniques in a young adult population that has been shown to be part of a higher-than-average food insecure community at the county and state levels. The percentage of the population that was food insecure in Brazos County in 2019 was 15.5%, higher than the Texas average of 14.1% [34]. The 2016 survey of the TAMU student body performed by the Everybody Eats project (*N*=1105) returned a rate of 40% self-identified at some level of food insecurity (9% food insecure, 31% sometimes food insecure) [35]. At a Land Grant University that is the flagship Agricultural and Extension institution in Texas, this is a particularly ironic problem. Integrating sustainable agricultural techniques and perspectives among this population is, we argue, crucial to both the future of food and the future of equitable human communities. Facilitating hands-on experience and understanding of the agricultural forms provides high-impact critical thinking to future leaders who will be making land use and food security decisions that will impact the survivability of humanity as a holistic ecological population.

The vast majority of Americans do not have a grounded understanding of where their food fundamentally comes from, nor of the political, economic, and ideological frameworks and practices that form and maintain the current food system. Even at TAMU students are not well-connected with information nor practices that provide their sustenance, nor with the already-arrived climate crisis that severely limits the sustainability of that sustenance. While TAMU and its larger regional community have several sustainability initiatives, they are not coordinated and integrated across the curriculum of any of its colleges, and regenerative agriculture is barely present at all. Regenerative agriculture is of importance here as it goes well beyond attempts to sustain a food system that contributes significantly to the degradation and loss of equitably accessible fertile soil and its necessary products. TAMU is a training ground for leadership and selfless service of the human community, but it struggles in the most fundamental arenas where such leadership and service are needed. Since everybody eats, it is imperative that the realities of maintaining soil fertility and health, maintaining potable water, and reaching towards zero waste are disseminated and learned in practical and easy-to-manage ways at the local, nay, household, residential level (see, e.g., [36]). Doing so from the center of an authoritative body whose stated duty to the public is to extend its publicly funded research is nothing more relevant than its very reason for existence.

### Research in the Time of COVID-19

Given that our research is ethnographic, and thus qualitative in nature, our methods of approach have been impacted. Despite COVID-19 restrictions, our research has continued, and there are indications that with appropriate mitigation practices, there may be even greater community interest in our food access facilitation model. For instance, the Southern Exposure Seed Exchange (SESE) catalog has noted that there is a direct uptick of desire to participate in the growing of food locally and residentially, and in March when orders typically decrease, orders kept doubling until it was necessary to limit orders [3]. SESE grows out their own seeds, but also works with other growers nationwide to provide seed, and both COVID-19 responses and climate change impacts (such as the wildfires across the western USA) converged to impact the open-pollinated seed supply regularly offered to the public. This example of a crucial supply chain also notes, “…most farmers make their crop plans during the winter, and they did not have the flexibility to take on much extra [seed crops] in the spring” [Ibid].

Given this increased interest, we piloted social-distanced windowsill herb gardens in the Fall of 2020. Participants were provided with a small box, soil, seeds, instructions, and easy to follow recipes in which to use their produce. While this pilot was small, it was promising and allowed for understanding of how participants and researchers, of many different backgrounds, interact and negotiate power. These participants will be followed over the winter and offered larger container gardens appropriate to their spatial contexts to practice growing more foods at home. The expertise of Aitkenhead-Peterson [37–40] and Brown [41–46] in terms of interaction of water and soil, rainwater use, and the development of urban microclimates will be brought to bear in assessing these residential gardens.

## Proposed Innovative Framework

We have four objectives:

1. Establish model micro-scale regenerative agriculture practices among on-campus students and residences in the community.
2. Integrate regenerative agriculture education as a form of civic agriculture across the University curriculum.
3. Build a model program that trains a cohort of students to think of themselves as farmers that produce and consume the products of regenerative civic agriculture.
4. Strengthen the pipeline of informed farmers, particularly those from underrepresented racial/ethnic/gender minority groups that have historically experienced dispossession and currently face food insecurity.

## Mechanisms for Bringing Together Intellectually Diverse Researchers and Stakeholders

Our primary strategy is to mobilize existing resources and expertise of the Land Grant/Cooperative Extension system to act as a regional hub for facilitating full community food security (caloric and nutritional adequacy) and food sovereignty (participatory decision-making regarding living spaces and culturally appropriate foodways). By gathering current, past, and new partners into an existing practice framework, a nexus of faculty provides a mechanism for bringing together and sustaining a community of intellectually diverse researchers and stakeholders. However, such a nexus must come from interdisciplinary backgrounds and take seriously the diversity of epistemological traditions of the academic and non-academic community participants across this project. Furthermore, this nexus must be able to leverage the breadth and depth of existing networks. To illustrate, since coming together specifically to answer the TAMU Convergence Research Incubator call to form working groups focused on some aspect of the Water-Food-Energy Nexus, we have committed to developing a collaboration as well as leveraging these ties across several related projects:

First, Gatson has led a longitudinal, collaborative research and service-learning effort in the local community since 2014 [TAMU IRB#2013-0764D]. The Everybody Eats project is a multilevel, interdisciplinary research-intensive community, wherein graduate students lead teams of undergraduates in researching different aspects of community food security. As well as being framed as participatory action research, the project is innovative as it integrates research, teaching, and service across formal and informal education/learning settings [47].

Second, Cisneros has established a Health Disparities track of the Biomedical Research Certificate in the Department of Veterinary Physiology & Pharmacology. In this track, she teaches predominately future health care providers. This track focuses on team-based undergraduate research development, particularly in the arena of nutrition education, access, and implementation.^3^ Prior to this, she worked as collaborator and research team leader with Gatson’s Everybody Eats project since 2015 and has expertise in sociology of food and culture, specifically food justice, food ways, and health and nutrition. Cisneros is part of a collaborative USDA-AFRI Research Grant (2018–2022, “Actionable Links between Soil Function, Ecosystem Services, and Stakeholder Perceptions to Overcome Barriers to Improved Soil Management”) as a research assistant.

Third, Brown has expertise in studying urban heat islands and analyzing and developing microclimates to mitigate them in the context of urban greenspace [41–45], inclusive of urban food crops and edible landscaping [46]. His recent work focuses on paying particular attention to the use of natural UV light in risk mitigation of COVID-19 spread while continuing to develop and deploy community-accessible greenspace and food gardening, in part using the container garden framework of Everybody Eats.

Fourth, Aitkenhead-Peterson has expertise in soil nutrients and farming strategies that avoids soil contamination common in urban environment [49]. She was involved in the TAMU Urban Farm United (TUFU) project at the outset as internship advisor to Broch Saxton along with Lisette Templin of the Dept. Health and Kinesiology from Texas A&M Office of Sustainability to start a project on aeroponic tower farming [50]. Based on the tower-garden project, she is currently working on establishing an urban agriculture certification for undergraduates, an online certification in urban agriculture for entrepreneurs and non-profits through extension, and research comparing soil and soil-less production of food in urban centers. She has published work on irrigation water chemistry, and its effect on soil nutrient status across urban centers in Texas [40] and on soil microbial community composition [38], soil nutrient chemistry for smallholder farms in Ghana [37], and plant uptake of Fluoride and heavy metals in small holder farms in southern India [39].

Fifth, Zhang brings her expertise in implementing several horticultural projects as well as studies in food economics and consumer research [51, 52]. She is currently conducting several survey studies related to food security during the COVID-19 outbreak, including consumer demand for home gardening especially edible plants, and the impacts of COVID-19 on people’s willingness to support food banks. Similar to curricular practices of the Everybody Eats project, she is teaching Honors AGEC105 (Introduction to Agricultural Economics), wherein around 15 undergraduate students in the University Honors Program will in part engage with such authentic research and service-learning projects in synergy with the frameworks used by Everybody Eats, particularly household/consumer-level engagement with food economies and supply chains.^4^

On the one hand, this nexus of faculty exemplifies the potential for leveraging diverse perspectives, lived experiences, disciplinary focus, and methodological expertise to address the complexities of a circular economy. On the other, this nexus also exemplifies the potential for leveraging a shared service-learning advocacy perspective embedded in a participatory action framework to develop strong ties and maintain sustainability of a common research program. Furthermore, members of this nexus have participated in and cultivated overlapping communities of practice that span conventional academic/community boundaries and have challenged researcher/researched dichotomies. By extending active networks outwards and leveraging “the strength of weak ties” [54], this nexus forms a model for recruiting intellectually diverse researchers and stakeholders and cultivating sustainable community that is both embedded and emerging.

## Potential Impact on Society

### Impact on Scholarship

From a macro-level perspective, the framework of agroecology takes a historical, anthropological, and sociological approach to understanding the historical development and ongoing everyday practices involved in human embeddedness in local ecologies, particularly those that result in distinctive foodways and food economies. We seek to better understand mechanisms leading to community food security, food justice, and food sovereignty by embedding the experiences and voices of micro-level food production and food access actors.

### Community-Level Impacts

We anticipate (1) a strengthened collaborative effort that takes advantage of the ways in which synergy among different disciplinary research agendas will move from parallel collegial interest to mutually constitutive resolution of social problems in the community; and (2) participatory and emancipatory sustained outreach to the community, wherein students, faculty, and non-academic community members collaborate in research extensive solutions to basic problems in fulfilling human and ecological needs.

### Individual-Level Impacts

We anticipate (1) practical, hands-on skill development across the food supply/provisioning chain: soil development (composting), selecting climatic, seasonal, and culturally appropriate food plants, planting/harvesting/processing such plants, and preparing meals from such plants; (2) a deep awareness of what is possible in current arrangements of the local ecological context, and how to acquire such an awareness upon moving to a new context; (3) a stronger connection to understanding the needs of one’s own household, as well as those of the larger community; and (4) a pathway to being empowered to act in service of fulfilling personal and communal needs on an equitable basis.

## Footnotes

1 For an excellent summary of these conditions during 2020, see “Dear Valued Customers & Gardening Friends & Everyone Everywhere” in the Southern Exposure Seed Exchange catalog [3].

2 See also Reese [6], in terms of the social organization involved in enacting food geographies at the household and community level; see McMillan [7] in terms of worker contexts involved in market arenas of provisioning: farm workers, supermarket employees, and restaurant cooks and waitstaff.

3 The work of Cisneros and students would be especially fruitful if engaged at the micro/household-level of the model suggested by Wirth et al. [48], with households seeing themselves as part of the nutrient food cycle/circular economies of nutrition.

4 Zhang’s work has the potential to engage household management in conversation with agribusiness management, introducing a robust cooperative economic framework to the practice of regenerative circular economy [53].
